# Supplementary material for: Legionella DotM structure reveals a role in effector recruiting to the Type 4B secretion system
Source: Nat Commun. 2018 Feb 6;9:507. doi: 10.1038/s41467-017-02578-x (PMC5802825; doi:10.1038/s41467-017-02578-x)
Supplement: Supplementary file 1 — Supplementary Information [file 41467_2017_2578_MOESM1_ESM.pdf]

Supplementary Information

Supplementary Figure 1. Superposition of wild-type, M1, M2, and M4 structures.

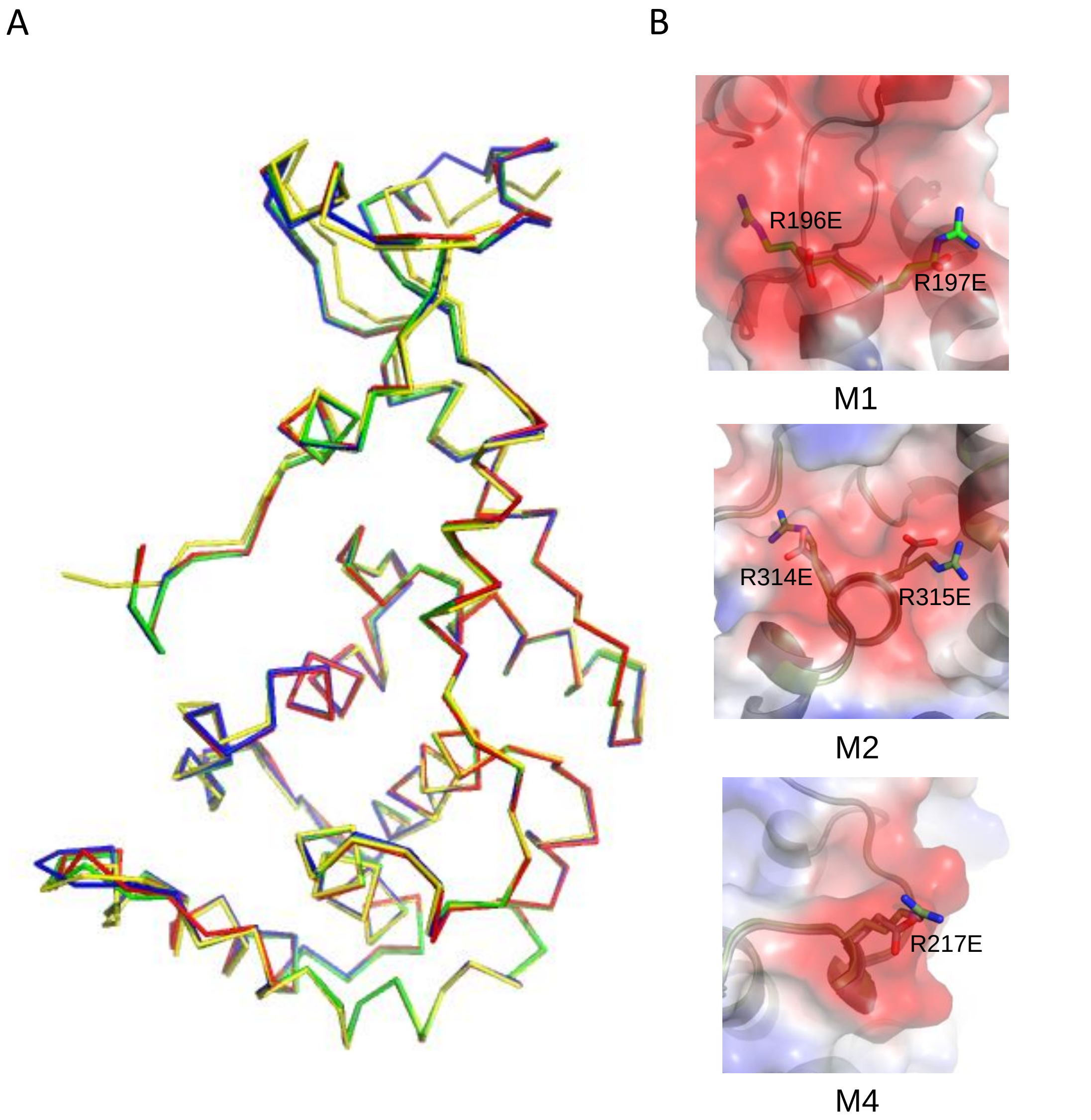

(A) Superposition of wild-type DotM153 structure (green) and its mutants M1 (blue), M2 (yellow) and M4 (red). The structures are shown in ribbon representation. The overall mutant structures are conserved, with minor root-mean-square deviation of 0.149, 0.311 and 0.194 Å, respectively, compared to the wild-type DotM153.

(B) Electrostatic surface representation of DotM153 mutants. Each panel shows the region where M1, M2, and M4 mutations where made. In each, the superposition of the

corresponding mutant and wild-type structures is shown with the backbone structure in cartoon representation and residues mutated in stick representation color coded as in Figure 1C.

Supplementary Figure 2. DotM and DotM mutants stability *in vivo* and translocation of full-length Cya-LegC8 fusion by *L.pneumophila* into host eukaryotic cells.

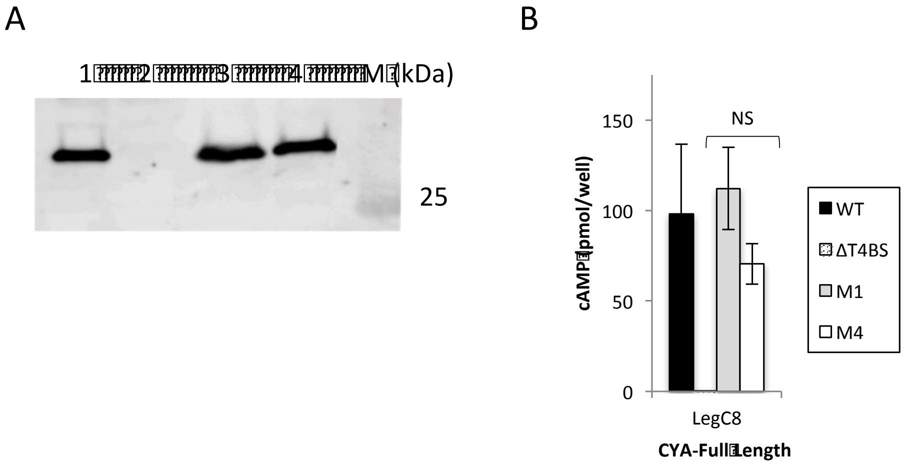

(A) DotM stability in *L.pneumophila* LP01 strains (WT, ΔT4BS, M1 and M4, numbered 1-4, respectively). Cells extracts were analysed by Western blotting using DotM specific antibody. M: molecular weight markers. Full gel figures are available in Figure S3.

(B) Translocation of the full-length Cya-LegC8 fusion by *L.pneumophila* into host eukaryotic cells as in Figure 6C.

Supplementary Figure 3. Full gels usesd in this study. Rectangles show the parts of the gels used in making the figures. The corresponding figures in which these parts are used are named under the rectangles.

A

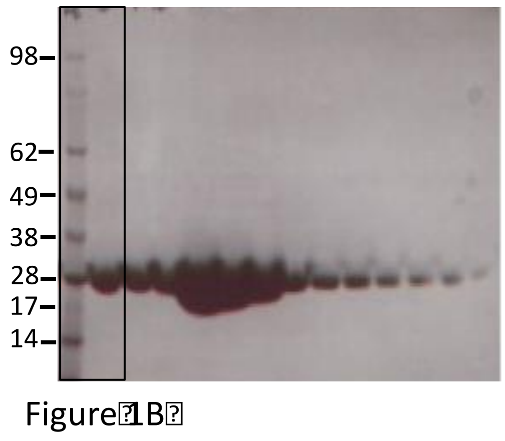

B

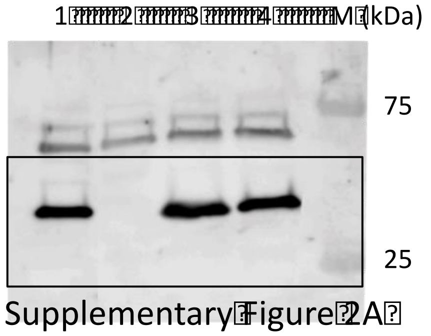

(A) Gel used to make Figure 1B.

(B) Gel used to make Figure S2A.

Supplementary Table 1: Strains, primers and plasmids used in this study

a. Strains used in this study

Strain	Description and Reference
***E.coli K12* strains used in this study**	
E.coli BL21(DE3) BLR	F^- *ompT hsdS*$_B$(r_B^- m_B^-) *gal dcm* (DE3) Δ(srl-recA)306::Tn*10* (Tet^R) (Merck milipore)
E. coli DH5α	*fhuA2 Δ(argF-lacZ)U169 phoA glnV44 Φ80 Δ(lacZ)M15 gyrA96 recA1 relA1 endA1 thi-1 hsdR17* (NEB)
E. coli DH5α λpir	sup E44, ΔlacU169 (ΦlacZΔM15), recA1, endA1, hsdR17, thi-1, gyrA96, relA1, λpir phage lysogen (Zuckman 1999)
E. coli CR019	MT607 *E. coli* containing plasmid pRK600; ColE1 replicon with RK2 transfer genes, Cm^R (Hubber 2014)
***Legionella pneumophila* strains used in this study**	
Lp01	$Strep^R$, *Legionella pneumophila* serogroup 1, Lp01 *rpsL*
ΔT4BS	$Strep^R$, Lp01 chromosomal deletions of three loci: *icmX–dotA*, *dotB–dotD*, and *icmT–dotU*
M1	$Strep^R$, Lp01 *dotM* R196E/R197E
M4	$Strep^R$, Lp01 *dotM* R217E
Lp01 $CegC3_{Cter}$	$Strep^R$, CM^R, Lp01 + pCYA-$CegC3_{Cter}$
Lp01 $Lpg1663_{Cter}$	$Strep^R$, CM^R, Lp01 + pCYA-$Lpg1663_{Cter}$
Lp01 OSM_{Cter}	$Strep^R$, CM^R, Lp01 + pCYA-OSM_{Cter}
Lp01 $LegC8_{Cter}$	$Strep^R$, CM^R, Lp01 + pCYA-$LegC8_{Cter}$
Lp01 $Lem21_{Cter}$	$Strep^R$, CM^R, Lp01 + pCYA-$Lem21_{Cter}$
Lp01 Cya	$Strep^R$, CM^R, Lp01 + pCYA
M1 $CegC3_{Cter}$	$Strep^R$, CM^R, M1 + pCYA-$CegC3_{Cter}$
M1 $Lpg1663_{Cter}$	$Strep^R$, CM^R, M1 + pCYA-$Lpg1663_{Cter}$
M1 OSM_{Cter}	$Strep^R$, CM^R, M1 + pCYA-OSM_{Cter}
M1 $LegC8_{Cter}$	$Strep^R$, CM^R, M1 + pCYA-$LegC8_{Cter}$
M1 $Lem21_{Cter}$	$Strep^R$, CM^R, M1 + pCYA-$Lem21_{Cter}$
M5 $CegC3_{Cter}$	$Strep^R$, CM^R, M5 + pCYA-$CegC3_{Cter}$
M5 $Lpg1663_{Cter}$	$Strep^R$, CM^R, M5 + pCYA-$Lpg1663_{Cter}$
M5 OSM_{Cter}	$Strep^R$, CM^R, M5 + pCYA-OSM_{Cter}
M5 $LegC8_{Cter}$	$Strep^R$, CM^R, M5 + pCYA-$LegC8_{Cter}$
M5 $Lem21_{Cter}$	$Strep^R$, CM^R, M5 + pCYA-$Lem21_{Cter}$
ΔT4BS $CegC3_{Cter}$	$Strep^R$, CM^R, M5 + pCYA-$CegC3_{Cter}$
ΔT4BS OSM_{Cter}	$Strep^R$, CM^R, M5 + pCYA-OSM_{Cter}
ΔT4BS $LegC8_{Cter}$	$Strep^R$, CM^R, M5 + pCYA-$LegC8_{Cter}$
**Eukaryotic cell lines used in this study**	
J774.1A	Macrophage from *Mus musculus* (ATCC TIB-67)
CHO FcγRII	Chinese hamster ovary expressing FcγRII (Nagai 2005)
Acanthamoeba castellanii	ATCC 30234

[a]antibiotic resistance: Cm, chloramphenicol; Strep, streptomycin

b. Plasmids used in this study

Construct	Description and Reference	Primers<br>Forward	 Reverse
pETM-41	Km^R; pBR322 (G.Stier)		
pETM-41Lin	Linearize pETM-41 with In-Fusion to yield N' [6His] open vector	PT001F	PT004R
pCC10	Km^R; [6His]DotM from *L.pneumophila* encoding residues 119-380 cloned into pETM-41Lin backbone (this study)	T4C12047F	T4C12043R
pCC10Lin	Linearize pCC10 with In-Fusion to yield N' [6Hispcs] open vector	PT001F	BD001R
pCC12	Km^R; CC10Lin with [6Hispcs]DotM from *L.pneumophila* encoding residues 119-371 (this study)	T4C12054F	T4C12055R
pCC16	Km^R; pCC10Lin with [6Hispcs]DotM from *L.pneumophila* encoding residues 153-380 (this study)	T4C12057F	T4C12043R
pCCP39	Km^R; pCC16 derivative encoding A R196E/R197E double mutation (this study)	T4C12076M1F	T4C12077M1R
pCCP41	Km^R; pCC16 derivative encoding A R314E/R315E double mutation (this study)	T4C12080M2F	T4C12081M2R
pCCP42	Km^R; pCC16 derivative encoding A R347E/R348E double mutation (this study)	T4C12082M3F	T4C12083M3R
pCCP43	Km^R; pCC16 derivative encoding A R217E mutation (this study)	T4C12093M4F	T4C12094M4R
pCCP44	Km^R; pCC16 derivative encoding A R262E mutation (this study)	T4C12095M5F	T4C12096M5R
pSR47S	Km^R; (J.J Merriam 1997)		
pSR47S-Lin	Linearize pSR47S with In-Fusion to yield an open vector	P47SF	P47SR
pSR47S-MI	Km^R; Full length DotM from *L.pneumophila* with 1000bp upstream and downstream cloned into pSR47S-Lin backbone (this study)	T4C12087CF	T4C12088DR
pSR47S-$DotM_{mut1}$	Km^R; pSR47S-MI derivative encoding A R196E/R197E double mutation (this study)	T4C12076M1F	T4C12077M1R
pSR47S-$DotM_{mut2}$	Km^R; pSR47S-MI derivative encoding A R314E/R315E double mutation (this study)	T4C12080M2F	T4C12081M2R
pSR47S-$DotM_{mut4}$	Km^R; pSR47S-MI derivative encoding A R217E mutation (this study)	T4C12093M4F	T4C12094M4R
pCYA	Cm^R;Cya domain cloned into pMMB207 (ATCC®37809) (Nagai 2005)		
pCYA-$CegC3_{Cter}$	Cm^R; Linearize pCya with last C-terminal 30 a.a. of [Cya]CegC3 from *L.pneumophila* (this study)	PCya002F	PCya001R
pCYA-$Lpg1663_{Cter}$	Cm^R; Linearize pCya with last C-terminal 30 a.a. of Lpg1663 from *L.pneumophila* (this study)	PCya003F	PCya001R
pCYA-OSM_{Cter}	Cm^R; Linearize pCya with last C-terminal 30 a.a. of OSM synthetic sequence (this study)	PCya005F	PCya001R
pCYA-$LegC8_{Cter}$	Cm^R; Linearize pCya with last C-terminal 30 a.a. of LegC8 from *L.pneumophila* (this study)	PCya008F	PCya001R
pCYA-$Lem21_{Cter}$	Cm^R; Linearize pCya with last C-terminal 30 a.a. of Lem21 from *L.pneumophila* (this study)	PCya007F	PCya001R

Pcs= HRV-3C protease cleavage site

[a]antibiotic resistance: Cm, chloramphenicol; Km, kanamycin; Strept, streptomycine.

c. Primers used in this study

**Primer for cloning**	
**Primer name**	**Primer sequence 5'-3'**
PT001F	taacaaagcccgaaaggaagctgagttg
PT004R	gtgatggtgatggtgatgtttcatggtatatctc
BD001R	ccctggaacagaacttccagggcgccgtgatggtgatggtgatgtttcatgg
P47SF	ggatcccccgggctgcaggaattcg
P47SR	ccactagttctagagcggccgcc
T4C12043	tcctttcgggctttgttatggctctaattcctccatttgacgtggtg
T4C12047	caccatcaccatcacgctaaatatcgcaaaacctatgatatgaaaagtttgcgg
T4C12054	gttctgttccaggggcccagcggcggtggcgcgaaatatcgcaaaacctatgatatgaaaagtttgcgg
T4C12055	tcctttcgggctttgttatgttaatctcacctcttttactgcaatttccag
T4C12056	cattatatctccttcttatgttaatctcacctcttttactgcaatttccag
T4C12057	gttctgttccaggggcccagcggcggtggcgcggacgtcaataaagggccttgggcaatggc
T4C12087CF	ctctagaactagtgggttgcaaaggctgttagctttaatcccggag
T4C12088DR	cagcccgggggatccgttactagttttggaatggtttcaatatgacg
**Primers for mutagenesis**	
**Primer name**	**Primer sequence 5'-3'**
T4C12076M1F	gagatgacagcggggatt**GAAGAA**ggcgatgccaaacgag
T4C12077M1R	ctcgtttggcatcgcc**TTCTTC**aatccccgctgtcatctc
T4C12080M2F	ggctcaaaccagtcgac**GAAGAA**ttatggtatatgttg
T4C12081M2R	caacatataccataa**TTCTTC**gtcgactggtttgagcc
T4C12082M3F	gaaaaggaaatggga**GAAGAA**tctttggtgccaatgatag
T4C12083M3R	ctatcattggcaccaaaga**TTCTTC**tcccatttcctttc
T4C12093M4F	ccttattgggatggttttgaa**GAA**tgctct cctcaggcttacgc
T4C12094M4R	gcgtaagcctgaggagagca**TTC**ttcaaaaccatcccaataagg
T4C12095M5F	gggaaacctgatttttctgttgca**GA**accagtaatgaaaaaataccaaaac
T4C12096M5R	gttttggtatttttcattactggt**TC**tgcaacagaaaaatcaggtttccc
**Primers for Cya constructs**	
**Primer name**	**Primer sequence 5'-3'**
PCya001R	cgcgccaccgccgctgtcatagccggaatcctggcgttcc
PCya002F	agcggcggtggcgcggaagaaaaagaagaagcagaattttcatcttctgaatcagaaaatgaagaaaaag aagaagaaaacgaagaatcaagccgttttacaatgtaactgcaggcatgcaagcttggctgttttg
PCya003F	agcggcggtggcgcgccctatgctgagcctaaagttgtatcagaagacaaagcggaatctgaagaagagaa cgaagatgaagagtcaagaaactcggcctcagtttaactgcaggcatgcaagcttggctgttttg
PCya005F	agcggcggtggcgcgttattattgttattatttagcagcgatgatgtgctgctggaagaagaagaagaagaaga agaaagcagcctgctgagcagcctgaaactgtaactgcaggcatgcaagcttggctgttttg
PCya007F	agcggcggtggcgcg ttgcaatatttgtccaggcattttacttcttctttaaataaactagcatctgttttagaagtgcaa ttgtttgccaacagat atgttccttagctgcaggcatgcaagcttggctgttttg
PCya008F	agcggcggtggcgcgata acttcctcagaagaagttgagcgtacacaatccctgcgaacggatggcttgagttggatgcctagtgagcaag cacgattatcaaaataactgcaggcatgcaagcttggctgttttg

bold: desired mutation
